# Supplementary material for: C24 Ceramide Lipid Nanoparticles for Skin Wound Healing
Source: Pharmaceutics. 2025 Feb 12;17(2):242. doi: 10.3390/pharmaceutics17020242 (PMC11859193; doi:10.3390/pharmaceutics17020242)
Supplement: Supplementary file 1 [file pharmaceutics-17-00242-s001.zip › pharmaceutics-3461863-supplementary.pdf]

## Supplementary Materials

# Ceramide-24 Lipid Nanoparticles for Skin Wound Healing

**Ji-Hye Lee [1*], Jin Hyun Kim [1*], Tong-Il Hyeon [1], Khee Tae Min [1], Seyoung Lee [1], Han-Chul Ko [1], Hong Seok Choi [1], Kuk-Youn Ju [1], Yung-Seok Cho [2#] and Tae-Jong Yoon [1,3,4#]**

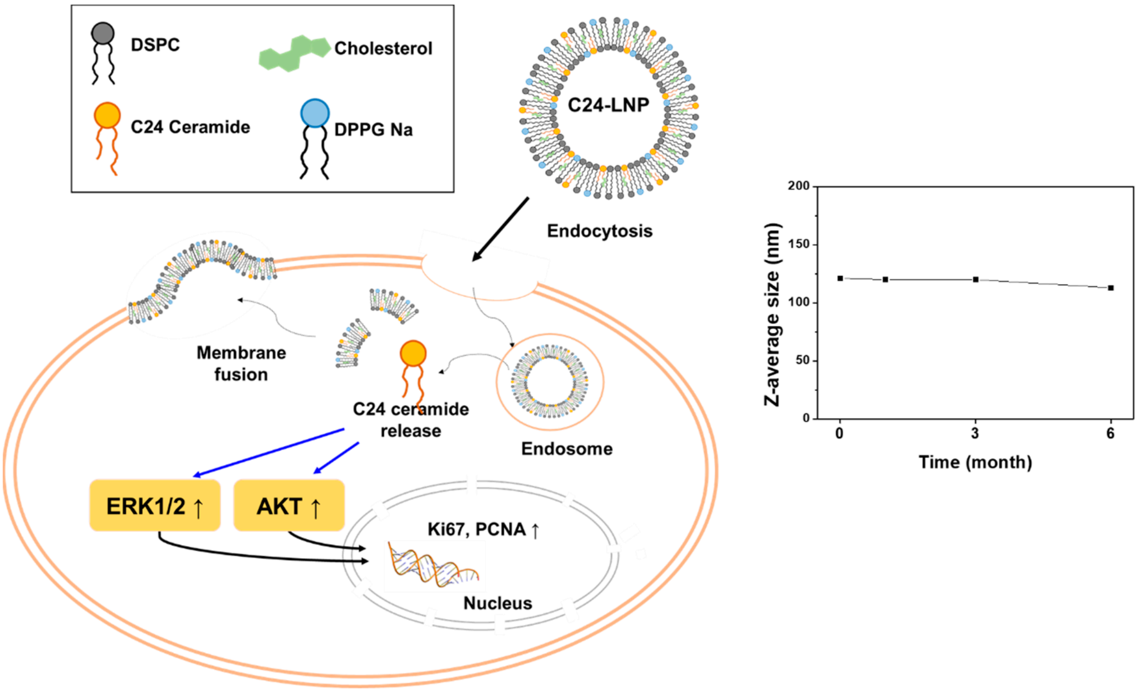


**Figure S1. Schematic showing the intracellular behavior of C24-LNP.** LNP enters cells through endocytosis, and C24 ceramide is exposed to the cytoplasm after endosomes are disrupted. Then, the main components of LNP are reabsorbed into the cell membrane, and the exposed C24 ceramide increases the expression of ERK1/2 and AKT in the cytoplasm. As a result, it promotes cell growth and migration (left). The synthesized C24-LNP showed solution stability for more than 3 months, confirmed through DLS measurement (right).

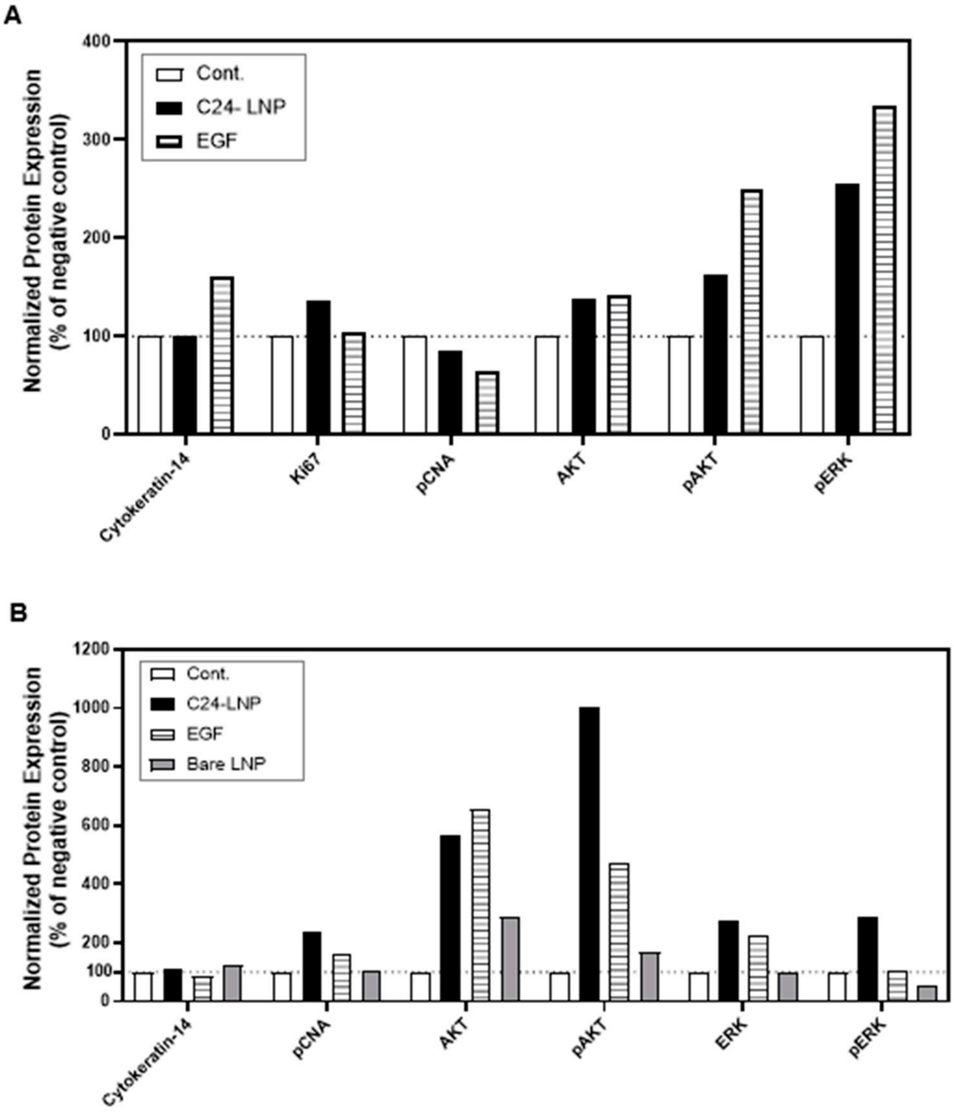


**Figure S2.** (A)Graph comparing the expression intensity of proliferation-related biomarkers in *in vitro* samples with untreated negative controls. (B) Analysis of proliferation-related signaling pathway biomarkers in HaCat cells. Strong activation was observed in samples treated with C24-LNP for 6 hours. (C) Data comparing the expression intensity of biomarkers in an *in vivo* animal model.

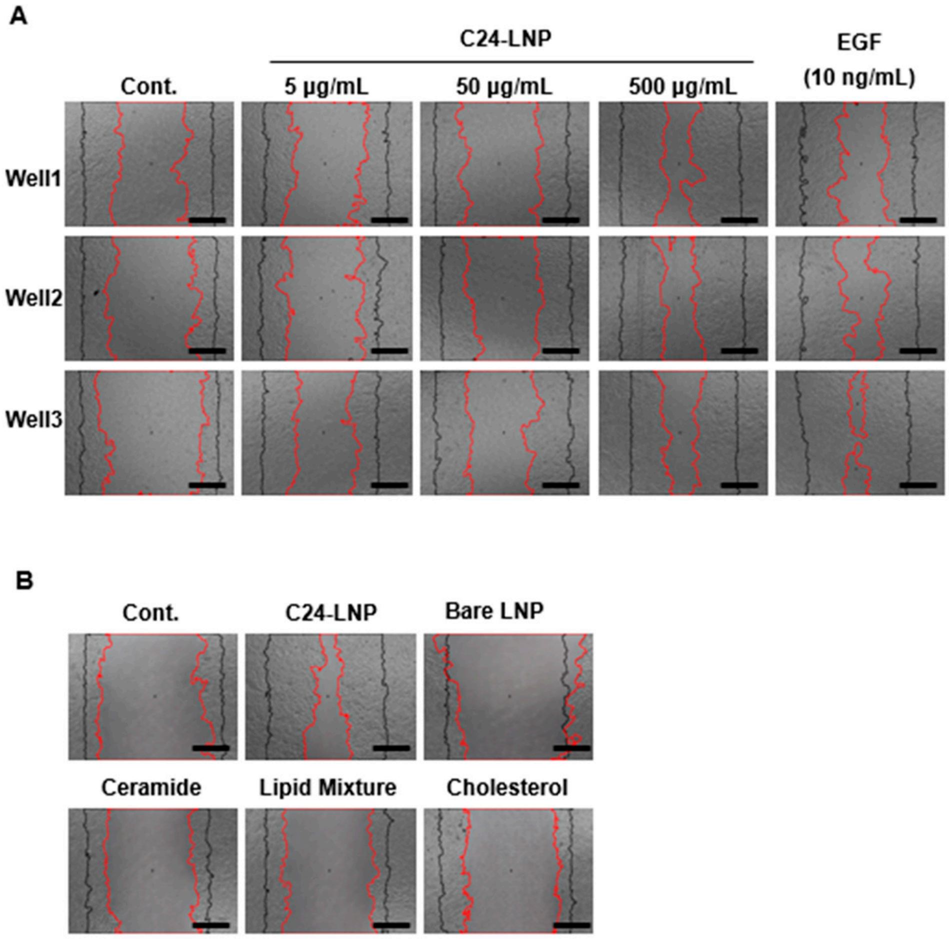


**Figure S3.** The *in vitro* migration effect was confirmed by observing the microscopic images according to the treatment concentration of C24-LNP. (A) Each sample was observed three times, and the black solid line indicates that the bottom of the cell culture well-plate was scratched. The red solid line indicates where the cells migrated after 48 hrs of continuous culture. (B) It was confirmed whether the cell migration effect increased when each component of C24-LNP was treated in the same way (100 µg/ml) . It was shown that the migration effect increased only when C24 was formulated as LNP. (Scale bar: 500 µm).

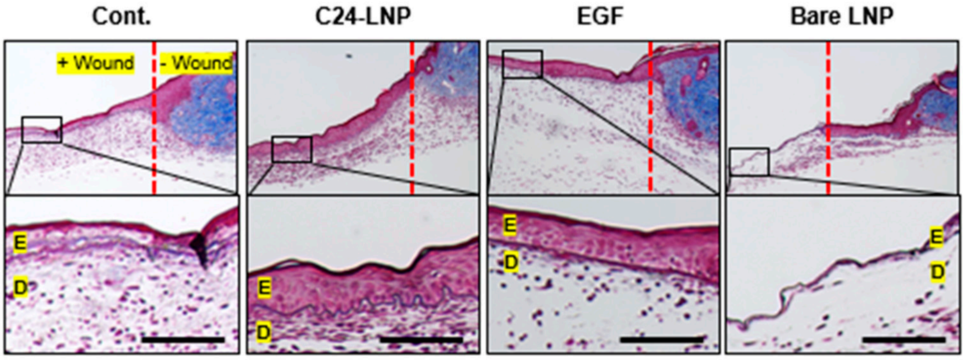


**Figure S4.** Images of histological analysis of collagen protein formation in the 4-day recovery group for various treatment groups, including C24-LNP. On the 4th day of wound healing, collagen formation in the dermis was observed to have not yet progressed. However, in the positive treatment group, the epidermis was observed to be very thick and showed rapid recovery. Tissues are H&E stained, and collagen proteins are shown in blue. (Scale bar: 100 μm).
